# Supplementary material for: Prevalence of latent tuberculosis infection among health workers in Afghanistan: A cross-sectional study
Source: PLoS One. 2021 Jun 1;16(6):e0252307. doi: 10.1371/journal.pone.0252307 (PMC8168887; doi:10.1371/journal.pone.0252307)
Supplement: Attachment — Submitted filename: Response to Reviewer #2.docx [file pone.0252307.s001.docx]

**Response to Reviewers’ Comments on “Prevalence of latent tuberculosis infection among health workers in Afghanistan: a cross-sectional study”**

**Reviewer #2**

We would like to thank Reviewer #2 for reviewing our article and providing valuable feedback. We have addressed the comments in the following way:

**1. Minor comment: The authors mention as two places that one third of the world population is suspected LTBI, however they also mention recent WHO data that show that number around one fourth (23%). Authors should reference and use the current data from WHO.**

We updated all the figures with the new WHO report, and actually the current TB infection prediction is 2 billion, which is around 26% of the world population in 2019.
